# Sense it and use it: interoceptive accuracy and sensibility in suicide ideators

**DOI:** 10.1186/s12888-019-2322-1

**Published:** 2019-11-01

**Authors:** Thomas Forkmann, Eftychia Volz-Sidiropoulou, Trientje Helbing, Barbara Drüke, Verena Mainz, Dajana Rath, Siegfried Gauggel, Tobias Teismann

**Affiliations:** 1 Department of Clinical Psychology, University of Duisburg-Essen, Essen, Germany; 2 Institute of Medical Psychology and Medical Sociology, University Hospital of RWTH Aachen University, Aachen, Germany; 3 Mental Health Research and Treatment Center, Department of Psychology, Ruhr-Universität Bochum, Bochum, Germany

**Keywords:** Suicide ideation, Subjective interoception, Objective interoception, Heartbeat perception, Interoceptive accuracy

## Abstract

**Background:**

Interoceptive deficits have been found to be associated with suicidal ideation and behavior. However, an objective measure of interoceptive accuracy has not been investigated in participants with suicide ideation, by now. This study aimed at investigating interoceptive accuracy and sensibility in persons with and without suicide ideation (SI) while controlling for severity of depressive symptoms.

**Method:**

Ninety-five participants (age: M = 34.8, SD = 11.6, *n* = 56 female [58.9%]; *n* = 51 patients with a Major Depressive Disorder and *n* = 44 healthy participants) were assessed for interoceptive accuracy and sensibility, depression and SI.

**Results:**

Twenty-five participants (26%) reported SI. They showed interoceptive accuracy comparable to persons without SI (*t* = −.81, *p* = .422), but significantly lower interoceptive sensibility. After controlling for severity of depressive symptoms in a hierarchical linear regression analysis, most associations between interoceptive sensibility and SI disappeared.

**Conclusion:**

Results suggest that suicide ideators do not lack the ability to perceive their own bodily signals but they feel less able to use them in a way that is advantageous for them. Differences between suicide ideators and non-ideators appear to be largely driven by depressive symptoms (depression bias).

## Introduction

Recent research suggests an association between interoceptive deficits on the one hand and suicide ideation and suicidal behavior on the other hand [1, 2]. Interoception is the ability to effectively perceive the physiological condition of the body [3, 4]. Garfinkel and Critchley [5] proposed to differentiate between *interoceptive accuracy*, i.e. the ability of perceiving physiological sensations, *interoceptive sensibility*, i.e. judgements of one’s dispositional ability to perceive body sensations, and *interoceptive awareness*, referring to an individual’s metacognitive awareness of his interoceptive accuracy. Recent research found that the three facets of interoception are related but yet distinct constructs [6–8]. Furthermore, Forkmann and colleagues [6] argued for the integration of a fourth facet of interoception, i.e. the ability to correctly monitor and evaluate physiological states, such as the individual’s heart rate, which is supposed to be the most basic level of interoceptive signal processing.

Forrest et al. [[2]; study I] compared interoceptive sensibility in controls, suicide ideators, suicide planners and attempters. In an online study, they found that those suffering from suicide ideation or behavior reported lower interoceptive sensibility than controls. Moreover, attempters stated lower interoceptive sensibility than planners or ideators. In a second study including psychiatric outpatients, self-reported interoceptive sensibility deficits were greater among those who attempted suicide compared to those who only thought about or planned suicide [[2]; study II]. Furthermore, Dodd et al. [1] provided evidence suggesting that impaired interoceptive sensibility is related to suicide attempts indirectly through mediating variables such as non-suicidal self-injury. These findings suggest that persons suffering from suicide ideation and behavior have greater difficulties of being in touch with their own bodily experiences. On this background, Forrest et al. [2] speculate that being “disconnected from one’s bodily self, facilitates suicide attempts for those who desire suicide” (p. 755).

However, both studies [1, 2] assessed interoceptive sensibility, i.e. subjective judgments of one’s dispositional tendency to be internally focused, using a rating scale and did not include a psychophysiological measure of interoceptive accuracy. Furthermore, both studies used an inconclusive self-report measure of interoceptive sensibility, the Interoceptive Awareness subscale of the Eating Disorder Inventory [9]. This scale is primarily targeted to assess two types of physiological sensations, emotional and gastrointestinal sensations, but less to measure interoceptive sensibility. Only recently, Rogers, Hagan and Joiner [10] used the Multidimensional Assessment of Interoceptive Awareness [MAIA [11];] to measure interoceptive sensibility more broadly in a large sample (*N* > 500) of adult participants with suicidal ideation/ behavior across the entire spectrum of the suicidality continuum. They found no differences in interoceptive sensibility between individuals with lifetime suicidal ideation, plans or attempts. Participants with lifetime suicidal ideation reported higher scores in worrying about body sensations than nonsuicidal participants. In addition, self-reported interoceptive deficits in terms of ignoring or distracting oneself from uncomfortable or painful body sensations and impaired self-regulation were larger in persons with lifetime suicide attempts compared to participants without a history of suicidal ideation/ behavior. Overall, participants with any history of suicidal ideation/ behavior trusted their bodily sensations less than nonsuicidal participants.

Although the study by Rogers and colleagues [10] provided a more detailed analysis of the relation between different aspects of interoceptive sensibility and suicide ideation/behavior, there has been no research on markers of interoceptive accuracy in terms of the performance in correctly sensing bodily sensations. Since prior research suggests that there are different facets of interoception, measured with different methodological approaches, it is possible that interoceptive accuracy relates differently to suicidal ideation and behavior than interoceptive sensibility. An interoceptive accuracy task allows for a more objective assessment of interoceptive performance deficits and might thus be a more suitable indicator of potentially impaired interoceptive processing in persons with suicidal ideation/ behavior than questionnaires.

Another important variable that has not been considered in prior investigations on the relationship between interoception and suicidal ideation/behavior is depression. Depression has also been shown to be related to interoception: People with elevated depression scores tend to have lower interoceptive accuracy [12–14]. Since suicidal ideation/ behavior and depression often co-occur [15], it is important to control for depression when investigating the relationship between suicidal ideation/ behavior and interoception. Only recently, a first study was published that controlled for depression while investigating the relation between interoception and suicidal ideation [16]. The reported results are mixed: when controlling for depression, only in two out of seven samples, a significant relation between interoceptive deficits and suicidal ideation were found.

Therefore, the aim of the present study was to assess interoceptive accuracy, using a heartbeat perception task, and interoceptive sensibility, using a comprehensive self-report measure while controlling for depression, in a heterogenous sample of subjects with or without current suicide ideation and with and without a Major Depressive Disorder (MDD). The results reported by Rogers et al. [10] suggest that differences in interoception should most likely be found between non-suicidal controls and participants with suicidal ideation, but not between participants with suicide ideation and those who attempted suicide. Thus, investigating interoceptive deficits in patients with suicide ideation compared to non-suicidal controls appears appropriate. In line with prior investigations, we expected persons with suicidal ideation to have lower interoceptive sensibility and accuracy than persons without suicidal ideation. Moreover, depression was expected to be related to both suicidal ideation and indicators of interoception.

## Methods

### Participants

The sample consisted of *N* = 95 participants (age: M = 34.8, SD = 11.6, range 18 to 55 years, *N* = 56 female [58.9%]), 51 of whom (age: M = 34.5, SD = 11.5, range 18 to 55 years, *N* = 32 female [62.7%]) suffered from a MDD according to the International Classification of Diseases, 10th edition (ICD-10) [17], and 44 (age: M = 35.2, SD = 11.8, range 18 to 54 years, *N* = 24 female [54.5%]) did not suffer from any mental disorder. Fourteen (27.5%) of the patients with a current depressive episode had 1–5 comorbid mental disorders: F34.1 (*n* = 4), F4x (*n* = 8), F5x (*n* = 6), F6x (*n* = 7), F7x (*n* = 0), F8x (*n* = 1), and F9x (*n* = 2). Patients were recruited from the Psychiatric Clinic of the University Hospital of the RWTH Aachen and three local psychiatric and psychosomatic hospitals. To be eligible for participation in the study, patients had to meet the criteria for a depressive episode at time of examination which was proofed by the International Diagnostic Checklist for ICD-10 [IDCL [18];]. Participants were excluded if they suffered from psychotic symptoms or an organic mental disorder, or if they were addicted to alcohol or drugs. Healthy participants were included if any acute mental disorder could be ruled out. Besides a general socio-demographic interview with a variety of health-related questions, inclusion criteria for healthy participants were checked by different rating scales: Screening-questions of the Structured Clinical Interview for ICD-10 [SCID [19];], Rasch-based Depression Screening [DESC-I [20];], and State-Trait-Anxiety Inventory [STAI [21];]. No participant reported any suicide attempts in their lifetime. The study has been approved by the local ethics committee (reference number EK 106/14) and was conducted in accordance with the declaration of Helsinki.

### Measures

#### Assessment of heart rate

The heart rate was recorded by means of the wrist-portable Polar V800 (1000 hz). This is a simple and valid way to record heart rate and interbeat intervals [22]. The V800 was applied to the participants and after 15 min of rest, a 7-min measurement of heart rate was carried out during which the participants sit quietly and relaxed on a comfortable chair. Heart rate was recorded for all healthy control participants in our laboratory in the same room. Patients’ heart rate was recorded either in the laboratory or in rooms provided by the respective hospital where they were treated. The recorded measuring section was further processed by means of the ARTiiFACT software [23] including artifact detection, removal and interpolation.

#### Heartbeat perception task

As a measure of interoceptive accuracy the Heartbeat Perception Task (HPT) was conducted similar to Schandry [24]. Participants were asked to monitor their heart beat and count the beats silently. They were not allowed to take their pulse and watches had to be removed beforehand. The task instruction was presented on a computer screen. Five trials of this task varying in length (24, 34, 44, 54, and 64 s) were performed, intermitted by short resting periods of 20 s between trials. A simultaneous visual and acoustical cue signaled the beginning and end of each trial. After each trial, participants were asked to indicate the number of perceived heartbeats by using the keyboard. They neither received feedback about their performance nor were they told the lengths of the counting phases. *Interoceptive Accuracy* was represented by the performance on the HPT, quantified by the heartbeat perception score [HPS [24];] with possible values from 0 to 1. The score was calculated with the formula: $HPS=\frac{1}{5}\ast \sum \left(1-\frac{\left| recorded\ heartbeats- perceived\ heartbeats\right|}{recorded\ heartbeats}\right)$. A value of 1 represents perfect accuracy.

#### Multidimensional assessment of interoceptive awareness

The Multidimensional Assessment of Interoceptive Awareness [MAIA [11];] consists of 32 items measuring different facets of self-reported interoceptive sensibility. Participants have to rate on a six-point Likert-scale ranging from 0 to 5 in how far they agree with each of the 32 statements.

The MAIA consists of eight subscales: “Noticing” (sample item: “When I am tense I notice where the tension is located in my body.”; Cronbach’s *α* in the current sample .56), “Not Distracting” (sample item: “When I feel pain or discomfort, I try to power through it.”; Cronbach’s *α* in the current sample .58), “Not Worrying” (sample item: “I can notice an unpleasant body sensation without worrying about it.”; Cronbach’s *α* in the current sample .54), “Attention Regulation” (sample item: “I can maintain awareness of my inner bodily sensations even when there is a lot going on around me.”; Cronbach’s *α* in the current sample .90), “Emotional Awareness” (“I notice how my body changes when I am angry.”; Cronbach’s *α* in the current sample .76), “Self-Regulation” (sample item: “When I bring awareness to my body I feel a sense of calm.”; Cronbach’s *α* in the current sample .87), “Body Listening” (sample item: “I listen to my body to inform me about what to do.”; Cronbach’s *α* in the current sample .80) and “Trusting” (sample item: “I trust my body sensations.“; Cronbach’s *α* in the current sample .91). High scores indicate high interoceptive sensibility in the respective domain.

#### Rasch-based depression screening

Depressive symptoms were assessed using the Rasch-based Depression Screening [DESC-I [20, 25, 26];]. The DESC-I comprises 10 items referring to the last two weeks, which are answered on a five point Likert scale ranging from 0 to 4. Internal consistency in the present sample was Cronbach’s α = .96. The suicide ideation item of the DESC-I was excluded from the measure for the present analyses to avoid artificially enhanced correlations with suicide ideation. Higher scores on the DESC indicate higher levels of severity of depressive symptoms.

#### Suicidal ideation

Current suicidal ideation was assessed with a single question taken from the DESC-I [20] asking the participants on a 5-point Likert scale ranging from “never” to “always”: “During the last two weeks, how often did you consider suicide as a potential way out?”. All participants who answered at least “seldom” to this question were considered as suicide ideators.

### Procedure

After a screening by phone, participants arrived at the laboratory room, were informed about the study and gave written informed consent to their participation. Patients with a depressive disorder were assessed either in the hospital in a quiet room or in the laboratory if possible. They were interviewed by an experienced researcher using the IDCL-checklist. Healthy participants answered the SCID-screening questions for mental disorders. All participants filled in the DESC and the STAI. Thereafter, if participants met the inclusion criteria, they were fitted with wrist-portable Polar V800, rested during the psychophysiological baseline measurement and then performed the HPT. The Sociodemographical Questionnaire and the MAIA were filled in after the HPT. All participants received the tests and questionnaires in the same fixed order. After approximately 1 hour they were thanked and paid 20 € for their participation.

### Statistical analyses

Means and standard deviations (SD) were calculated for all study variables, and separately for patients with a depressive disorder and healthy controls, and for participants with suicide ideation and those without. Means of measures of depression, interoceptive accuracy and sensibility and heart rate were compared between groups using t-tests for independent samples. In addition, effect sizes and confidence intervals were calculated. To control for the effect of depression severity on the relation between interoception and the frequency of suicide ideation, three multivariate hierarchical linear regression analyses were calculated. In all linear regression analyses, depression severity was entered in the first step and measures of interoceptive accuracy (first analysis) and interoceptive sensibility (second analysis) in the second step. Predictors were checked for multicollinearity prior to analyses (variance inflation factor (VIF) < 5.0 and tolerance > 0.2 for all predictors). All analyses were conducted using IBM SPSS Version 25 for Windows. Effect sizes (Hedges g) and 95% confidence intervals (95% CI) were calculated with the EffectSizeCalculator (https://www.cem.org/effect-size-calculator).

## Results

### Descriptive statistics

Table 1 shows descriptive statistics of patients with a depressive episode and healthy controls. Twenty-two (43.1%) of the patients with a MDD and three (2.3%) of the healthy controls reported suicide ideation in the past two weeks (*χ*^*2*^ = 16.07, *p* < .001).

**Table 1:** sample description

	depressed patients (*n* = 51)					controls (*n* = 44)										
	N	%	M	SD	Range	N	%	M	SD	Range	*t*	*p*	*χ* ^*2*^	*p*	*ES* ^c^	*95% CI*
Gender (female)	32	62.7				24	54.5						0.30	0.58		
Age			34.5	11.5	18–55			35.2	11.8	18–54	0.29	0.77			−.06	[−.46, .34]
Suicidal ideation	22^a^	43.1^a^	0.7	1.0	0–4	3^a^	2.3^a^	0.1	0.4	0–2			16.07	0.00	.76	[.34, 1.18]
Depressive severity			18.9	8.1	1–36			2.8	3.5	0–15	−12.60	<.01			2.50	[1.96, 3.03]
HPS^b^			0.7	0.2	0.4–0.9			0.7	0.2	0.3–1.0	0.01	1.00			.00	[−.40, .40]
Noticing			3.0	0.9	1.3–5.0			3.2	0.9	1.3–4.8	1.30	0.20			−.22	[−.62, .18]
Not Distracting			1.7	0.9	0.0–3.7			2.2	0.9	0.7–4.7	3.09	<.01			−.55	[−.96, −.14]
Not Worrying			2.2	1.1	0.7–5.0			2.7	0.9	0.7–4.3	2.72	0.01			−.49	[−.90, −.08]
Attention Regulation			2.1	1.0	0.1–4.6			3.0	0.8	1.6–4.7	4.82	<.01			−.98	[−1.40, −.55]
Emotional Awareness			3.2	0.9	1.4–4.6			3.4	0.8	1.6–4.8	1.19	0.24			.84	[−.64, .17]
Self Regulation			1.7	1.0	0.0–3.8			3.0	1.0	0.8–5.0	6.17	<.01			−1.29	[−1.73, −.85]
Body Listening			1.4	1.0	0.0–3.3			2.5	1.0	0.7–4.0	5.32	<.01			−1.09	[−1.52, −.66]
Trusting			1.9	1.3	0.0–5.0			3.9	1.1	1.7–5.0	7.84	<.01			−.1.64	[−2.10, −1.17]
Heart rate			77.9	11.9	58.8–105.2			76.1	12.0	57.1–98.2	−0.68	0.50			.15	[−.25, .55]

Note: ^a^at least “seldom”; ^b^HPS: Heartbeat Perception Score; ^c^effect size Cohen’s d; numbers in bold are significant at *α < .05*

### Differences in measures of interoception between suicide ideators and non-ideators

**Fig. 1:**
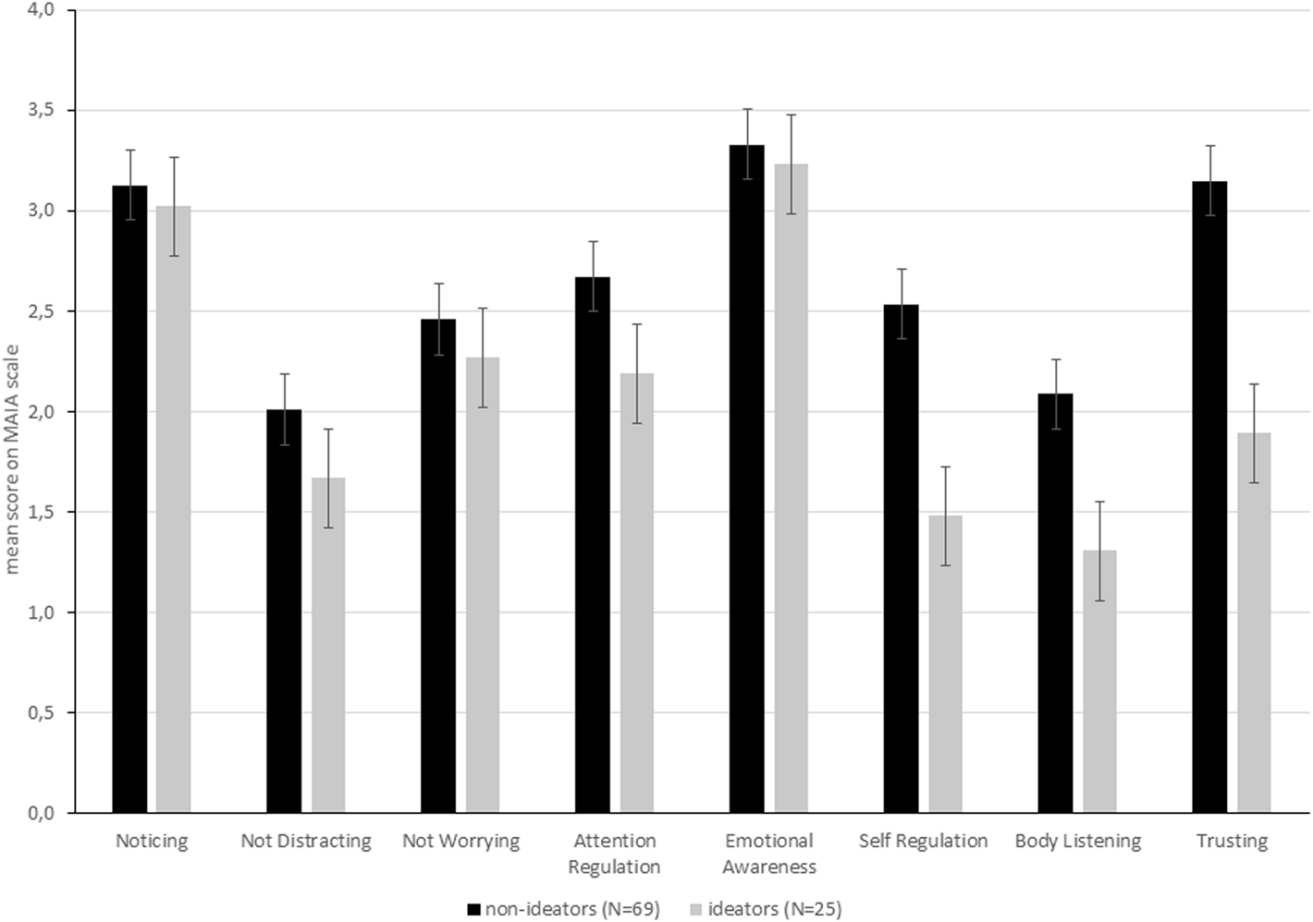
Differences in measures of interoceptive sensibility between suicide ideators and non-ideators

Using independent samples t-tests (Table 2 and Fig. 1) to compare mean interoceptive accuracy (HPS) between suicide ideators and non-ideators revealed no significant differences (t = −.81, *p* = .422, Hedges g = .00, 95% CI [− 0.46, 0.46]). However, interoceptive sensibility differed between groups. Suicide ideators reported significantly lower levels on the following scales: attention regulation (t = 2.1, *p* = .037, Hedges g = .50, 95% CI [0.03, 0.96]), self-regulation (t = 4.0, *p* < .001, Hedges g = .92, 95% CI [0.45, 1.40]), body listening (t = 3.2, *p* = .002, Hedges g = .77, 95% CI [0.30, 1.24]), and trusting (t = 3.8, *p* < .001, Hedges g = .85, 95% CI [0.38, 1.32]). There was no difference in mean heart rate between the groups (t = −.94, *p* = .35, Hedges g = −.25, 95% CI [− 0.71, 0.21]), but a significant difference with a large effect size in depression severity (t = 7.97, *p* < .001, Hedges g = − 1.85, 95% CI [− 2.37, − 1.32]).

**Table 2:** differences in interoceptive accuracy, sensibility, heart rate and depression severity separated between suicide ideators and non-ideators

	non-ideators (*n* = 69)		ideators (*n* = 25)					
	M	SD	M	SD	t	p	ES^a^	95% CI
HPS_global	0.7	0.2	0.7	0.1	− 0.81	0.42	.00	[−.46, .46]
Noticing	3.1	0.9	3.0	0.9	0.52	0.61	.11	[−.35, .57]
Not Distracting	2.0	0.9	1.7	0.9	1.65	0.10	.33	[−.13, .79]
Not Worrying	2.5	1.0	2.3	1.1	0.79	0.43	.19	[−.27, .65]
Attention Regulation	2.7	1.0	2.2	1.0	2.11	0.04	.50	[.03, 0.96]
Emotional Awareness	3.3	0.8	3.2	0.8	0.51	0.61	.12	[−.33, .58]
Self Regulation	2.5	1.1	1.5	1.0	4.04	< 0.01	.92	[.45, 1.40]
Body Listening	2.1	1.1	1.3	0.8	3.23	< 0.01	.77	[.30, 1.24]
Trusting	3.1	1.4	1.9	1.4	3.77	< 0.01	.85	[.38, 1.32]
Mean heart rate	76.3	11.0	79.4	14.8	−0.93	0.35	−.25	[−.71, .21]
DESC sumscore (without suicide)	7.3	7.8	21.4	6.9	−7.97	< 0.01	−1.85	[−1.85, −1.32]

*Note: HPS: Heartbeat Perception Score.* ^*:^ *Effect size Cohen’s d; numbers in bold are significant at α < .05*

### Hierarchical linear regression analyses on the relation between measures of interoception and suicide ideation

Table 3 shows correlations between all variables that were entered in the hierarchical linear regression analyses. Regression analyses investigating the relation between interoceptive accuracy and suicide ideation revealed that, when controlling for depressive symptoms, depression (β = .57, *p* < .001) but not interoceptive accuracy (β = .12, *p* = .22) were significantly related to suicide ideation (Table 4). When repeating this analysis adding measures of interoceptive sensibility (MAIA), again, depression was significantly related to suicide ideation (β = .74, *p* < .001). Above, only the MAIA scale “not worrying” was significantly related to suicide ideation (β = .23, *p* = .03).

**Table 3:** Correlations between all variables entered in the hierarchical linear regression analyses

	Noticing	Not Distracting	Not Worrying	Attention Regulation	Emotional Awareness	Self Regulation	Body Listening	Trusting	Interoceptive Accuracy	Suicide Ideation
Noticing										
Not Distracting	.10									
Not Worrying	−.02	.03								
Attention Regulation	**.36**	**.25**	.17							
Emotional Awareness	**.56**	**.24**	.06	**.39**						
Self Regulation	**.27**	**.34**	**.27**	**.60**	**.42**					
Body Listening	**.35**	**.48**	.20	**.62**	**.53**	**.62**				
Trusting	**.28**	**.41**	.19	**.69**	**.37**	**.69**	**.72**			
Interoceptive Accuracy	−.05	−.12	.17	.08	−.04	−.00	−.13	−.01		
Suicide Ideation	−.14	**−.22**	.07	−.18	−.09	−.**38**	**−.27**	**−.37**	.12	
Depression	−.09	−.**32**	**−.29**	**−.49**	−.13	**−.60**	**−.53**	**−.72**	.04	**.57**

Note: numbers in bold are significant at *α < .05*

**Table 4:** Results of hierarchical linear regression analyses predicting suicide ideation

	Model 1				Model 2				Model 3			
	B	β	t	p	B	β	t	p	B	β	t	p
Intercept	−0.11		−1.08	0.28	−0.48		−1.52	0.13	−1.46		−2.80	0.01
Depression	0.04	0.57	5.93	< 0.001	0.04	0.57	5.99	< 0.001	0.05	0.74	4.95	< 0.001
Interoceptive accuracy (HPS)	–	–	–	–	0.53	0.12	1.23	0.22	0.29	0.06	0.65	0.52
Noticing	–	–	–	–	–	–	–	–	−0.03	−0.04	− 0.31	0.76
Not Distracting	–	–	–	–	–	–	–	–	0.09	0.12	1.09	0.28
Not Worrying	–	–	–	–	–	–	–	–	0.16	0.23	2.30	0.03
Attention Regulation	–	–	–	–	–	–	–	–	0.12	0.17	1.24	0.22
Emotional Awareness	–	–	–	–	–	–	–	–	0.12	0.14	1.13	0.26
Self Regulation	–	–	–	–	–	–	–	–	−0.08	−0.13	−0.94	0.35
Body Listening	–	–	–	–	–	–	–	–	−0.11	− 0.16	−0.95	0.35
Trusting	–	–	–	–	–	–	–	–	0.08	0.17	0.91	0.37
	R^2^ = .33				R^2^ = 0.34				R^2^ = .43			
Model	Adj. R^2^ = .32				Adj. R^2^ = .32				Adj. R^2^ = .34			
	F= 35.15				F = 18.45				F = 4.88			
	*p* < .001				*p* < .001				*p* < .001			
Change in R^2^	0.33				0.01				0.94			
	*p* < .001				*p* = .222				*p* = .251			

Note: *HPS* Heartbeat Perception Score

## Discussion

The present study is, to the best of our knowledge, the first that investigated both interoceptive accuracy and sensibility while controlling for depressive symptoms in suicide ideators and healthy controls. Results suggest that suicide ideators are as competent in sensing their bodily signals as non-ideators when assessed with a psychophysiological measure of interoceptive accuracy. Suicide ideators report lower values in some indicators of interoceptive sensibility (MAIA) than non-ideators. However, these differences disappear when regression analyses were controlled for depressive symptoms. Moreover, when controlling for depression in a multivariate linear regression analysis it emerged that suicide ideators tend to worry more about their body sensations than non-ideators.

Using the same instrument (i.e., MAIA) to assess interoceptive sensibility as in the present study, Rogers et al. [10] found that individuals with lifetime suicide ideation reported more worry about their bodily sensations than people without a lifetime history of suicidality. People with lifetime suicide attempts tended to ignore and distract themselves more from painful or uncomfortable bodily sensations than non-suicidal participants. Participants with any kind of suicidality (regardless of whether reported lifetime ideation, plans, or attempts) reported less trust of their bodily sensations. There were no differences in the other scales of the MAIA. In the present study, we investigated people with suicide ideation in the past 2 weeks in comparison to non-suicidal controls. Thus, the present sample is not fully comparable to the suicide ideators sample in the study by Rogers et al. [10]. However, similarly to Rogers et al. [10], we found a difference between non-ideators and ideators in the extent of reported trust in their own body - although both groups showed similar performance in an interoceptive task (i.e., HPT). Those participants who reported suicide ideation in the past 2 weeks trusted their bodily signals less. Moreover, ideators reported to be less able to sustain and control attention to body sensations, to regulate distress by attention to body sensations, and to listen actively to the body in order to gain more insight. Notably, all these aspects of interoceptive sensibility do refer to the ability to act on one’s own sensations in order to regulate attention or distress and not to the ability to gather information from one’s own body [11]. This partly corresponds to results from Rogers et al. [10] and the HPT results measuring interoceptive accuracy in the present study: both in terms of interoceptive accuracy and interoceptive sensibility, suicide ideators appear to be able to sense their bodily signals as well as non-ideators. However, in the MAIA (as a measure of interoceptive sensibility) they report that they are less able to act on them or use them functionally to regulate distress, which, ultimately, coincides with impaired body trust. Low body trust most likely leads to non-use of information from the body, an assumption that is corroborated by the result that suicide ideators report less body listening than non-ideators.

A considerable line of research suggests that access to and usage of information from the body is associated with better performance in memory [27], learning [28], and attention tasks [29], less depression [14, 30], more adaptive cardio-vascular responses to stress [31], fewer difficulties in self-reported and objective decision-making [32, 33], and, by trend, with less brooding rumination [34]. Consequently, impaired body trust and little listening to the body may coincide with deficits in these variables. The Integrative Motivational-Volitional Model of Suicide [IMV [35, 36];] proposes that memory deficits and biases, problem-solving deficits and perseverative thinking may contribute to the formation of suicidal thoughts and plans. Empirical evidence supports the main predictions of the IMV-model [37, 38]. Thus, our results of low self-reported body trust and body listening, which probably leads to deficient use of available interoceptive information from the body, may be seen as in line with the assumptions of the IMV-model: deficient use of interoceptive information might lead to problems in decision-making, problem-solving, and memory and to heightened rumination and, in turn, contribute to the development of suicide ideation. Of course, this line of reasoning should be further investigated in future studies.

The results that suicide ideators reported lower abilities to sustain and control attention to body sensations and to regulate distress by attention to body sensations compared to non-ideators may indicate a potential mechanism contributing to the development and maintenance of suicide ideation. Recent research suggests that people with suicide ideation benefit from Mindfulness-based Cognitive Therapy [MBCT [39–42];], which combines cognitive-behavioral elements such as psychoeducation with meditation. Thus, people with suicide ideation benefit from an intervention that teaches them to deliberately direct attention to body sensations and to *use* body sensations (especially one’s own breath) to regulate their state of mind. This might indirectly be interpreted as suggesting that impaired abilities to sustain and control attention to body sensations and to regulate distress by attention to body sensations contributes to the development and maintenance of suicide ideation. Future research could address this issue more directly.

Table 2 shows that, *generally,* participants with suicide ideation reported lower levels of abilities than participants without suicide ideation. Group differences could also be considered as reflecting a general tendency of suicidal persons to be less self-confident than non-suicidal persons: suicidal persons could tend to ascribe themselves low capabilities, regardless of what concrete ability they might be asked for. Research showing that suicidal ideation/ behavior is related to low self-confidence could be considered as being in line with this interpretation [43].

However, when appreciating these results, it is of utmost importance to keep in mind that most differences between ideators and non-ideators vanished when controlling for depression. Thus, differences between suicide ideators and non-ideators could be overshadowed by a depression bias. Future research should aim at replicating the current findings and at investigating whether potential deficits in interoceptive sensibility are driven by heightened depression severity alone. Moreover, studies are lacking that investigate the interoceptive *awareness* which has not been studied in people with suicidal ideation/ behavior at all [5, 6].

### Limitations

Some strengths and weaknesses of the current study have to be kept in mind when appreciating the reported results. This is the first study that investigated a measure of interoceptive accuracy in suicide ideators. Results were controlled for depression and the participants reported reasonable divergent levels of depression severity. A limitation is that suicide ideation was assessed with a single item instead of a more comprehensive method to assess suicide ideation. Yet, there is strong evidence for the predictive ability and relevance of single items assessing suicide ideation [44]. Second, no suicide planners or attempters were included in the present investigation. Although prior research found no differences between suicide ideators, planners and attempters in terms of interoceptive sensibility [10] these patients could likely have differed in terms of interoceptive accuracy. Future research should aim at replicating our findings in a sample covering the entire spectrum of suicidality. Third, the present study and all prior studies on the relation between interoception and suicidality were cross-sectional [1, 2, 10]. However, the cross-sectional design limits the interpretation of the results as no causal conclusions can be drawn. Future studies should apply prospective designs in order to clarify whether interoceptive deficits are a risk factor for the development of suicidal ideation and behavior, contribute to its maintenance, or are a consequence of a suicidal development. Fourth, all participating patients were assessed in the hospitals where they were treated. As measurements had to fit in the schedule of the respective units where the patients were treated, unfortunately, it was not possible to control for room temperature and time of the day for the physiological assessments. Lastly, some scales of the MAIA had poor internal consistency in the current sample. Thus, reliability of assessments with these scales was limited.

## Conclusions

Taken together, results suggest that suicide ideators do not lack the ability to perceive their own bodily signals but they do not use them properly. They report less interoceptive sensibility suggesting that they use this information less, in terms of a reduced ability to regulate body-related attention or use body sensations for distress regulation. Group differences depended on depression severity. Future research could use prospective designs to investigate causal relations between interoception and suicidality and could consider potential interactive effects of depression and interoception on suicidal ideation and behavior.

## Data Availability

All relevant data are reported within the paper. Raw data are available from the corresponding author on reasonable request.

## Abbreviations

95% CI: 95% confidence intervals
DESC-I: Rasch-based Depression Screening
HPS: Heartbeat Perception Score
HPT: Heartbeat Perception Task
ICD-10: International Classification of Diseases, 10th edition
IDCL: International Diagnostic Checklist for ICD-10
IMV: Integrative Motivational-Volitional Model of Suicide
MAIA: Multidimensional Assessment of Interoceptive Awareness
MBCT: Mindfulness-based Cognitive Therapy
MDD: Major Depressive Disorder
SCID: Structured Clinical Interview for ICD-10
SD: Standard deviation
SI: Suicide ideation
STAI: State-Trait-Anxiety Inventory
VIF: Variance inflation factor
